# Accuracy of a prototype dark blood late gadolinium enhancement technique for the detection and quantification of myocardial infarction

**DOI:** 10.1186/1532-429X-18-S1-Q65

**Published:** 2016-01-27

**Authors:** Akos Varga-Szemes, Giuseppe Muscogiuri, Wolfgang G Rehwald, Uwe Joseph Schoepf, Sheldon E Litwin, Carlo N De Cecco, Julian L Wichmann, Stefanie Mangold, Damiano Caruso, Stephen R Fuller, Pal Suranyi

**Affiliations:** 1 grid.259828.c0000000121893475Division of Cardiovascluar Imaging, Department of Radiology and Radiological Science, Medical University of South Carolina, Charleston, SC USA; 2 grid.7841.aDepartment of Medical-Surgical Sciences and Translational Medicine, University of Rome "Sapienza", Rome, Italy; 3 Siemens Healthcare, Chicago, IL USA; 4 grid.26009.3d0000000419367961Cardiovascular Magnetic Resonance Center, Duke University, Durham, NC USA; 5 grid.259828.c0000000121893475Division of Cardiology, Department of Medicine, Medical University of South Carolina, Charleson, SC USA; 6 Department of Diagnostic and Interventional Radiology, University Hospital Frankfurt, Frankfurt, Germany; 7 grid.10392.390000000121901447Department of Diagnostic and Inverventional Radiology, Eberhard-Karls University Tuebingen, Tuebingen, Germany; 8 grid.7841.aDepartment of Radiological, Oncological and Pathological Sciences, University of Rome "Sapienza", Rome, Italy

## Background

Conventional inversion recovery (IR) techniques for the detection of late gadolinium enhancement (LGE) in the myocardium are bright blood methods in which the signal in the blood chamber is often similar to the signal in hyperenhanced irreversibly damaged myocardial areas. Due to the low contrast to noise ratio (CNR) between blood and the hyperenhanced myocardium at the tissue-blood interface, the discrimination of subendocardial LGE from blood is often challenging with this approach. The aim of our study was to evaluate the accuracy of a prototype dark blood LGE technique for the detection and quantification of myocardial LGE in patients with myocardial infarction (MI) when compared to conventional IR LGE technique.

## Methods

Twenty-one patients (56 ± 15 years, 15 male) with suspected MI were prospectively enrolled and consented for a cardiac MRI on a 1.5T scanner (MAGNETOM Avanto, Siemens AG, Erlangen, Germany). Short and long-axis conventional bright-blood LGE imaging was performed 10-15 minutes post-contrast (0.1 mmol/kg gadobenate-dimeglumine, MultiHance, Bracco, Princeton NJ) using a single-shot steady-state free precession sequence with an IR pulse (FOV 380 mm, slice thickness (ST) 8 mm, matrix 114 × 192, TE/TR 1.1/2.6 ms, flip angle 50°, TI 230-320 ms, and effective TR ~1600 ms). Consequently, corresponding single-shot images were acquired using the prototype dark blood T(Rho) And Magnetization Transfer (MT) and Inversion Recovery ("TRAMINER") sequence (equivalent FOV, ST, matrix, TE/TR, and effective TR, flip angle 55°, and TI 130-150 ms). Image evaluation was performed according to the 17-segment model by two readers. To ensure correct detection of contours, corresponding cine images were reviewed. The volume and the global percentage of MI (sum of segmental transmurality scores divided by 17) were calculated and compared using the Wilcoxon test. Inter-reader agreement and diagnostic accuracy were calculated using κ and McNemar statistics, respectively.

## Results

A total of 357 myocardial segments were evaluated. Twelve segments (3.4%) were excluded from the analysis due to image artifacts. No significant difference was observed in MI volume (4.1 ± 1.7 vs. 4.4 ± 1.9 ml, *P* = N.S.) and global percentage of MI (10.9 ± 8.7 vs. 11.3 ± 7.1%, *P* = N.S.) between the conventional LGE and the TRAMINER technique. Inter-reader agreement was good (κ = 0.72) to excellent (κ>0.81) for the detection of hyperenhancement by LGE and TRAMINER. The sensitivity and specificity of TRAMINER for the detection of MI were 95.9% and 97.2%, respectively.

## Conclusions

In this study, the dark blood TRAMINER technique performed similarly to conventional LGE imaging. Considering its high sensitivity and specificity, the TRAMINER approach is a non-inferior alternative technique for the detection of MI. Additionally, due to its dark blood property, we hypothesize TRAMINER to be more sensitive towards the detection of small subendocardial MIs which may appear equivocal in conventional LGE imaging.

**Figure 1:**
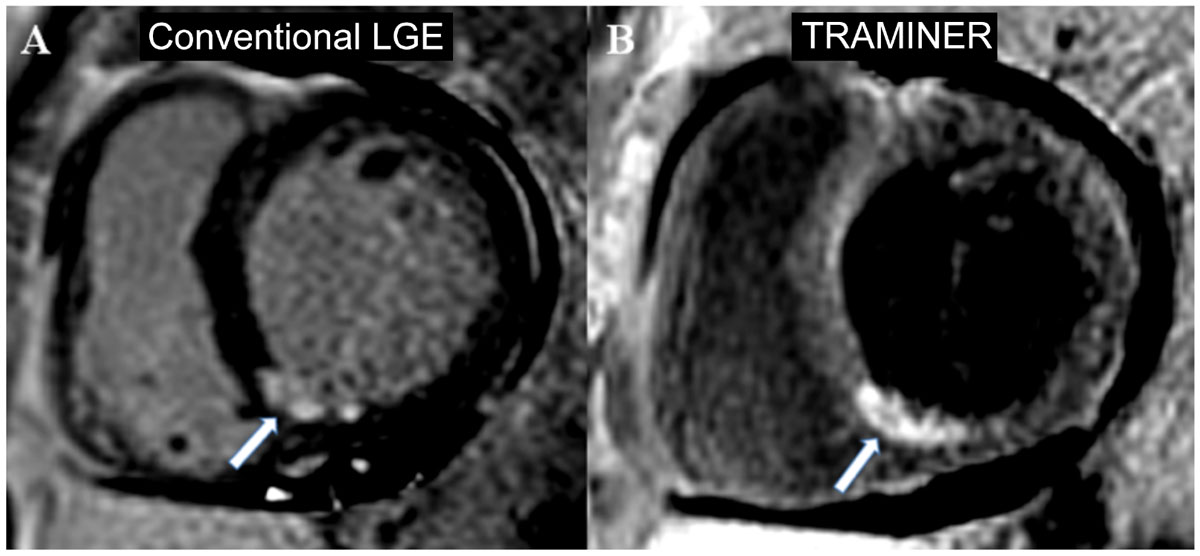
**Representative conventional bright blood (A) and prototype dark blood (B) IR images**.

